# A retrospective analysis on the utility and complications of upper arm ports in 433 cases at a single institute

**DOI:** 10.1007/s10147-015-0917-1

**Published:** 2015-10-27

**Authors:** Yukiko Mori, Satoshi Nagayama, Jun-ichiro Kawamura, Suguru Hasegawa, Eiji Tanaka, Hiroshi Okabe, Megumi Takeuchi, Makoto Sonobe, Shigemi Matsumoto, Masashi Kanai, Manabu Muto, Tsutomu Chiba, Yoshiharu Sakai

**Affiliations:** Department of Clinical Oncology, Kyoto University Hospital Cancer Center, 54 Shogoin Kawahara-cho, Sakyo-ku, Kyoto, 606-8507 Japan; Division of Gastrointestinal Surgery, Department of Surgery, Kyoto University Hospital, 54 Shogoin Kawahara-cho, Sakyo-ku, Kyoto, 606-8507 Japan; Department of Breast Surgery, Kyoto University Hospital, 54 Shogoin Kawahara-cho, Sakyo-ku, Kyoto, 606-8507 Japan; Department of Thoracic Surgery, Kyoto University Graduate School of Medicine, Kyoto University, Yoshidahonmachi, Sakyo Ward, Kyoto, Kyoto 606-8501 Japan; Department of Gastroenterology and Hepatology, Kyoto University Hospital, 54 Shogoin Kawahara-cho, Sakyo-ku, Kyoto, 606-8507 Japan

**Keywords:** Upper arm central venous ports, Long-term availability, CVP-related complications

## Abstract

**Background:**

We have employed upper arm central venous ports (UACVPs) since 2006 for long-term intravenous chemotherapy (CTx) or fluid supplementation. We evaluated the long-term availability of CVPs implanted in the upper arm to determine whether UACVPs could be one of the treatment options besides chest CVPs in terms of CVP-related complications.

**Methods:**

We reviewed the medical records of all patients who underwent subcutaneous implantation of UACVPs at Kyoto University Hospital from 1 April, 2006 to 30 June, 2009. We assessed the indwelling duration of the UACVPs and the incidences of early and late UACVP-related complications.

**Results:**

A total of 433 patients underwent subcutaneous implantation of UACVPs during this time period. The cumulative follow-up period was 251,538 catheter days, and the median duration of UACVP indwelling was 439.0 days (1–2, 24). There was no UACVP-related mortality throughout the study period. A total of 83 UACVP-related complications occurred (19.2 %), including 43 cases of infection (9.9 %, 0.17/1000 catheter days), ten cases of catheter-related thrombosis (2.3 %, 0.040/1000 catheter days), ten cases of occlusion (2.3 %, 0.040/1000 catheter days), nine cases of catheter dislocation (2.0 %, 0.036/1000 catheter days), five cases of port leakage (1.2 %, 0.019/1000 catheter days), four cases of skin dehiscence (0.9 %, 0.015/1000 catheter days) and two cases of port chamber twist (0.5 %, 0.008/1000 catheter days). The removal-free one-year port availability was estimated at 87.8 %.

**Conclusions:**

UACVPs were of long-term utility, with complication rates comparable to those of chest CVPs previously reported.

## Introduction

Central venous ports (CVPs) are good medical devices to facilitate the long-term administration of intravenous chemotherapy (CTx) or fluid supplementation. Since continuous systemic chemotherapy such as FOLFOX or FOLFIRI in combination with molecular-targeted drugs has been regarded as the standard treatment for advanced colorectal cancers [1–3], the subcutaneous implantation of CVPs has become an essential device in daily medical care. Moreover, since the combination regimen of 5-fluorouracil, folinic acid, oxaliplatin and irinotecan (FOLFIRINOX) has been proposed as a new standard of care for metastatic pancreatic cancer patients [4], the subcutaneous implantation of CVPs has been performed more frequently than ever before.

The most common site for the implantation of CVPs is the anterior chest via the subclavian vein (chest CVPs). However, the insertion of CVPs into the subclavian vein is sometimes complicated by pneumothorax, pneumohemothorax, or arterial punctures [5–8]. In addition, the long-term usage of chest CVPs is sometimes complicated with pinch-off syndrome, a severe complication in which the catheter becomes kinked, compressed or even fragmented at the narrow space between the clavicle and the first rib due to repetitive arm motion [9–11]. The implantation of CVPs via the internal jugular vein is considered to be safer compared to access via the subclavian vein [12–14]. In contrast, some authors prefer to implant CVPs in the upper arm or forearm via the basilic or axillary veins because of safer puncture procedures, and concluded that arm CVPs could be suitable for long-term usage with minimal complications [15–17]. Therefore, we chose to employ upper arm CVPs (UACVPs) rather than chest CVPs to prevent the possible complications associated with chest CVPs [18] and hypothesized that UACVPs could be one of the feasible options for CVPs, especially for systemic chemotherapy. In order to re-evaluate the utility of UACVPs, we examined UACVP-associated complications and long-term utility of UACVPs in a larger cohort of our patients.

## Patients and methods

**Fig. 1:**
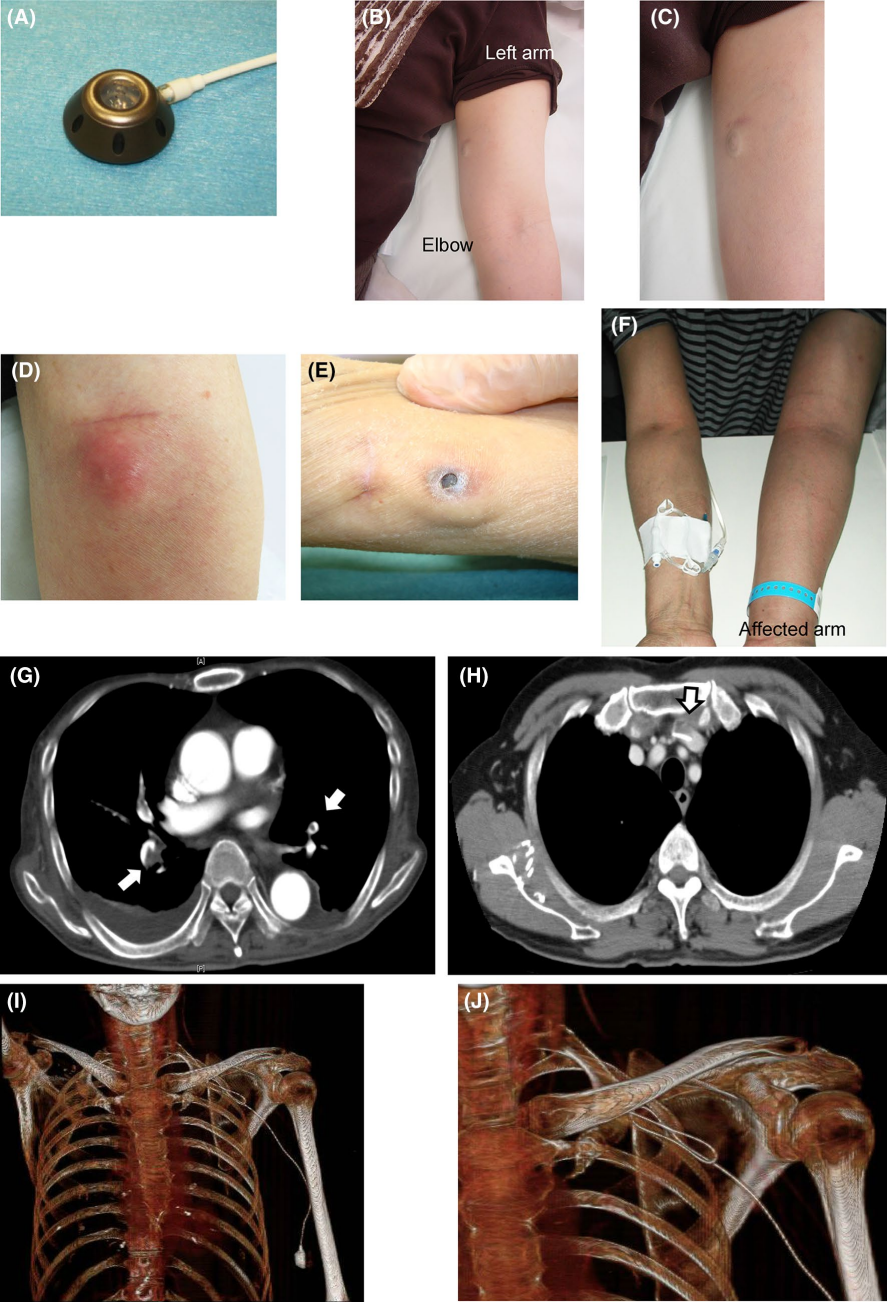
Subcutaneously implanted UACVP and representative manifestations of UACVP-related complications. **a** Titanium Vital Port system. **b**, **c** CVP was implanted on the ulnar side in the upper arm. **d** Port pocket infection with erythematic induration. **e** Skin dehiscence leading to the exposure of a subcutaneous CVP. **f** Venous thrombosis resulting in swelling of the left upper extremity. The patient underwent systemic anticoagulant therapy via the right peripheral blood vessel. **g** Bilateral pulmonary embolism. **h** Thrombus detected around the catheter in the subclavian vein. **i**, **j** Catheter dislocation on three-dimensional computed tomography

We reviewed the medical records of all patients who underwent subcutaneous implantation of UACVPs at Kyoto University Hospital from 1 April, 2006 to 30 June, 2009. UACVPs were implanted in 433 consecutive patients for the long-term administration of chemotherapy or fluid supplementation during this period, according to the implantation techniques described previously [18]. Almost all the patients (427, 98.6 %) suffered from malignant diseases including colon cancer (235, 54.3 %), gastric cancer (45, 10.4 %), breast cancer (45, 10.4 %), lung cancer (29, 6.7 %), pancreatic cancer (19, 4.4 %), esophageal cancer (18, 4.2 %) and other malignancies (36, 8.3 %). The majority of these patients (386, 89.1 %) required the implantation of UACVPs for systemic CTx (CTx group). For the remaining 47 patients, the UACVPs were implanted for fluid supplementation, or because of inaccessibility to the peripheral blood vessels (non-CTx group). Furthermore, 38 patients from the non-CTx group were at the end stage of their malignancies, and required best supportive care. We performed routine computed tomography scanning every 3 or 4 months in all patients from the CTx group to assess the effectiveness of the treatment, and to detect UACVP-related complications including venous thrombosis. Using the same implantation protocol guided by ultrasonography (US) [18], all procedures were performed by senior surgeons under local anesthesia in a day surgery unit equipped with a mobile X-ray fluoroscopic scanner and US device. The UACVPs were implanted in the patient’s non-dominant upper arm, with the exception of breast cancer patients with axillary lymph node dissection, who underwent UACVP implantation in the unaffected upper arm. In 2006, we employed a SlimPort^®^ system (Bard Access Systems, Salt Lake City, UT, USA), but later decided to employ a Titanium Vital Port^®^ system (Cook Vascular, Leechburg, PA, USA)(Fig. 1a) since the latter port is smaller and seems to be more suitable for subcutaneous implantation in the upper arm. We followed the patients with implanted UACVPs until March 2014. Follow-up was discontinued upon removal of the UACVPs, at the patient’s death, or at the end of the study period. The information was retrieved from the medical charts, and the data included UACVP-related infections, i.e., catheter infection and port pocket infection (Fig. 1d)], skin dehiscence (Fig. 1e), venous thrombosis (Fig. 1f–h), catheter dislocation (Fig. 1i, j) or occlusion, port chamber twist, port leakage, catheter fracture and needle dislodgement. Catheter infection was defined by the following conditions—(1) when blood culture tests were positive for microorganisms including a coagulase-negative staphylococcus (a typical pathogen associated with long-term indwelling of venous catheters), or (2) when blood culture tests were negative but a high fever persisted without any possible infection foci other than the CVPs. Port pocket infection was defined as an erythematic or painful induration, or tenderness at the port site often complicated with pus retention. Venous thrombosis was confirmed by enhanced computed tomography, magnetic resonance imaging or US. Catheter occlusion was defined as the failure to aspirate and flush the contents via CVPs. In this study, aspiration occlusion, where the blood cannot be aspirated but the port/catheter system can be flushed without resistance, was not regarded as catheter occlusion, since aspiration occlusion can be episodic and normal catheter function can recover spontaneously without any intervention [18]. The study was conducted in accordance with the Helsinki Declaration, and the protocol was approved by the Ethics Committee of Kyoto University Hospital (Approval No. E-845).

### Statistical analysis

The time to the development of UACVP-related complications and to port removal, i.e., complication-free and overall (removal-free) port availability, respectively was evaluated using the Kaplan−Meier product limit method. Patient death and port removal without any UACVP-associated complications, including scheduled removal at the cessation of chemotherapy, were analyzed as censored cases. Differences in UACVP-related complication rates by the addition of bevacizumab (Bmab) were evaluated using Fisher’s exact test. Statistical significance was assumed to be *p* < 0.05. All statistical analyses were performed using JMP version 9.0.0 (SAS Institute Inc., USA).

## Results

**Fig. 2:**
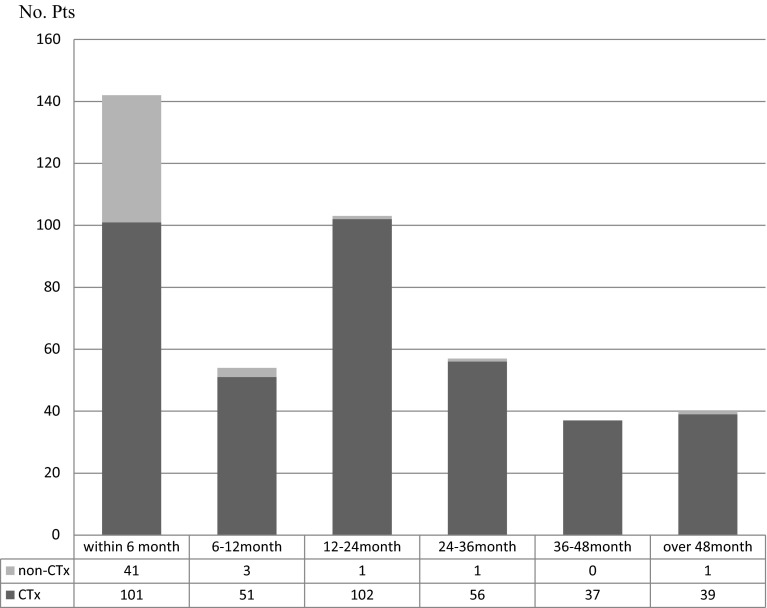
Indwelling periods of UACVPs according to the purpose of implantation. The black and gray bars represent the number of patients in the CTx and non-CTx groups, respectively. The median indwelling period was much shorter in the non-CTx group [22.0 (1–2047) days] compared to the CTx group [499.0 (1–2824) days]

**Fig. 3:**
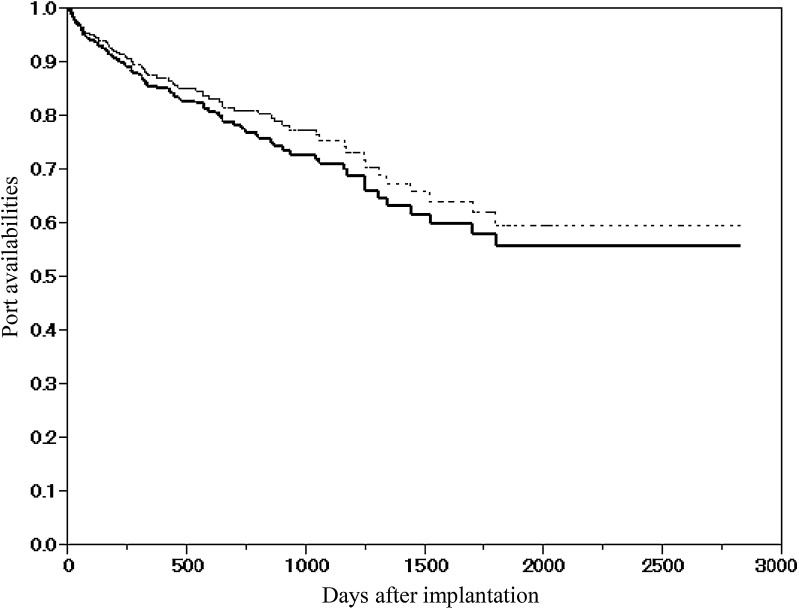
Complication-free 1-year port availability (solid line) and the overall one-year port availability (dotted line) in all patients were evaluated by the Kaplan–Meier method. The complication-free 1-year port availability was estimated at 86.0 %, whereas the overall 1-year port availability in the whole group was estimated at 87.7 %

A total of 433 ports were implanted within five attempts without any complications including pneumothorax, hemothorax, arterial puncture, or cardiovascular problems. The median indwelling period was 439.0 days (1–2824), the cumulative follow-up period was 251,538 catheter days, and the median event free period was 422.0 days (1–2824) (Table 1). The median indwelling periods were 499.0 (1–2824) and 22.0 (1–2047) days in the CTx and non-CTx groups, respectively. In 87.2 % (41/47) of patients in the non-CTx group, the indwelling periods were <6 months. In contrast, 60.6 % (234/386) of patients in the CTx group could use UACVP for more than a year (Fig. 2). In the CTx group, 74 (19.2 %) patients received systemic CTx including Bmab, a recombinant humanized monoclonal antibody directed against vascular endothelial growth factor, and the cumulative follow-up period and the median indwelling periods were 77,141 catheter days and 983.0 days (85–2810), respectively. The complication-free one-year port availability in all groups and the CTx group only was estimated at 86.0 and 87.3 %, respectively, whereas the overall one-year port availability in all groups and the CTx group only was estimated at 87.7 and 88.7 %, respectively (Fig. 3).

**Table 1:** Patient background

Characteristics	
Total (*n*)	433
Patient gender	
Female	186
Male	247
Age (years) (Mean ± SD)	63.39 ± 11.0
Primary disease	
Malignancy	427
Benign	6
Purpose of the implant	
Chemotherapy	386
Other	47
Median duration of implant (range days)	439.0 (1–2824)
Total duration of implant (days)	251,538

There was no UACVP-related mortality throughout this study. Of the 142 patients with an indwelling period <6 months, 47 out of 101 patients in the CTx group and 31 out of 41 patients in the non-CTx group died due to deterioration of their primary disease without any UACVP-related complications. During the long-term follow-up, UACVP-related complications occurred in a total of 83 patients (19.2 %) (Table 2). UACVP-related infections were evident in 43 patients (9.9 %, 0.17/1000 catheter days), and 27 of those patients were diagnosed with catheter infection (6.2 %, 0.11/1000 catheter days). A blood culture test was positive for bacteria in seven patients (1.6 %, 0.03/1000 catheter days) and for fungi in four patients (0.9 %, 0.02/1000 catheter days), but was negative in 12 patients. Of these 12 negative patients, a culture test for the catheter was also negative in 10 patients, but had not been performed in the remaining two patients. The UACVPs were removed in all patients suspected of catheter infection, who all recovered following the appropriate anti-bacterial and/or anti-fungal treatment. Port pocket infection occurred in 16 patients (3.7 %, 0.064/1000 catheter days), and was treated by port removal in ten patients and by administration of broad-spectrum antibiotics in the other two patients. In 44 patients from the non-CTx group for fluid supplementation, catheter and port pocket infections were evident in 3 (6.8 %) and 2 (4.5 %) patients, respectively. In contrast, in the 389 patients from the CTx group, 24 (6.2 %) and 14 (3.6 %) patients developed catheter and port pocket infections, respectively. There were no significant differences in infection rates (catheter and port pocket) between the non-CTx and CTx groups (*p* = 0.80).

**Table 2:** CVP-related complications

Complications	*n*	Complication/1000 catheter days (complication rate; %)	Evulsion	Treatment for the patients without evulsion		
				Antibiotic	Anticoagulant	Observation
Catheter infection	27	0.107 (6.2)	27	0	0	0
Port infection	16	0.064 (3.7)	14	2	0	0
Thrombosis	10	0.040 (2.3)	3	0	7	0
Obstruction	10	0.040 (2.3)	9	0	0	1
Catheter dislocation	9	0.036 (2.1)	9	0	0	0
Reserver leak	5	0.020 (1.2)	4	0	0	1
Skin complication (exposure)	4	0.016 (1.0)	4	0	0	0
Port rotation/flip	2	0.008 (0.5)	2	0	0	0
Total	83		72	2	7	2

Of the ten patients with venous thrombosis (2.3 %, 0.04/1000 catheter days), three complained of thrombosis-related symptoms (swelling of the upper extremities, shoulder pain and respiratory distress), and systemic anticoagulant therapy was initiated promptly with a gradual amelioration of their symptoms. Asymptomatic thrombosis was detected in five patients on a regular computed tomography examination performed every 3 or 4 months. In addition, concomitant, asymptomatic pulmonary embolism was confirmed radiographically in two of these five patients. All thrombotic patients underwent systemic anticoagulant therapy, and following the disappearance of the thrombi by the appropriate therapy, the UACVPs were removed in three patients who had no need for further chemotherapy. Of the 74 patients receiving Bmab-containing CTx, UACVP-related complications occurred in 22 patients (28.9 %, 0.29/1000 catheter days), including nine cases of UACVP-related infections (seven catheter infection and two port pocket infection) (12.2 %, 0.12/1000 catheter days), three cases of venous thrombosis (4.1 %, 0.04/1000 catheter days) and four cases of catheter occlusion (5.4 %, 0.052/1000 catheter days). Although the frequency of CVP-related complications tended to be higher in the Bmab-containing CTx group compared to the Bmab-free group (*p* = 0.022), there were no significant differences in the incidences of venous thrombosis between the two groups (*p* = 0.4119).

Of the nine patients with catheter dislocations (2.1 %, 0.036/1000 catheter days), two complained of chest or back pain at the initiation of routine CTx, whereas the remaining seven patients were asymptomatic, and the catheter dislocation was detected by a regular computed tomography examination. Port leakage was evident in five patients—the system was removed in four patients, and the other patient complained of mild swelling at the port pocket after each administration of CTx, possibly due to minimal leakage of the infusion. Since the patient’s performance status was unsatisfactory and there were no signs of infection, we did not replace it with a new system, and the cause of the port leakage remained unclear in this case. There were no cases of detectable fibrin sheath formation, catheter fracture or needle dislodgement. In a total of 118 patients including 47 patients whose UACVPs were removed due to completion of chemotherapy, 31 (26.3 %) developed catheter infection, which was the main cause leading to port removal due to complications (Table 2).

**Fig. 4:**
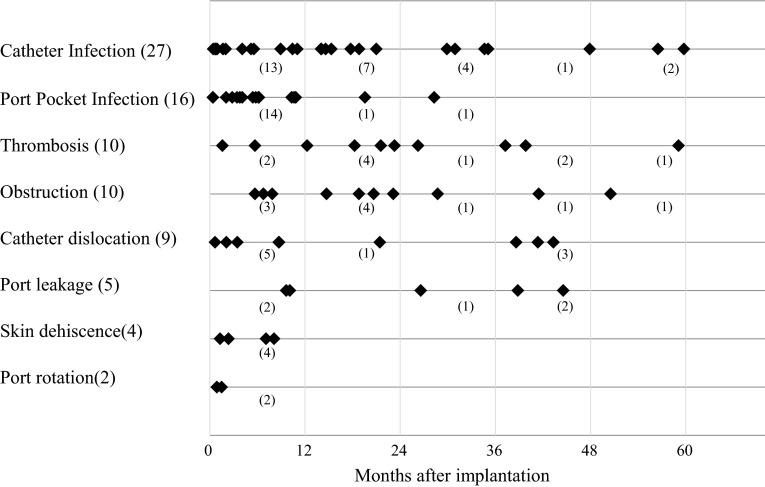
Indwelling periods at the onset of UACVP-related complications. Among the 74 patients with indwelling periods >3 years, 13 developed UACVP-related complications. UACVP-related infection tended to occur more frequently within one year after UACVP implantation, whereas venous thrombosis and catheter occlusion occurred regardless of the indwelling period. The parentheses in the bottom line indicate the number of patients with complications with UACVPs during the indicated period

With regard to the indwelling period at the onset of the UACVP-related complications (Fig. 4), port pocket infection, skin dehiscence and port leakage occurred within 1 year after UACVP implantation. Although half of catheter infection cases (13/27, 48 %) were evident within 1 year after UACVP implantation, seven patients developed catheter infection >2 years after implantation. In contrast, venous thrombosis and catheter occlusion occurred regardless of the indwelling period.

## Discussion

Although there are some arguments regarding the implantation site for CVPs, we have adopted UACVPs rather than chest CVPs because of the easier and safer access to the central blood vessel, as supported by Marcy et al. [16]. In this study, the UACVPs were placed successfully in all patients without any implantation-related complications, including pneumothorax, hemothorax, arterial puncture, or cardiovascular problems. The rates of UACVP-related complications including infection, thrombosis, dislocation and occlusion in this study were comparable to those of chest and forearm CVPs and other UACVPs reported previously [6, 7, 19–27] (Table 3). Consistent with a previous report describing US-guided implantation and the feasibility of UACVPs [18], we confirmed the safety of the implantation procedures and the long-term utility of UACVPs in a larger cohort of patients.

**Table 3:** Reported complication rates with chest and arm ports

	Type of port	No. of patient	Infection/1000 catheter days (complication rate; %)	Thrombosis/1000 catheter days (complication rate; %)	Migration/1000 catheter days (complication rate; %)	Occlusion/1000 catheter days (complication rate; %)	Port removal
Shetty et al. (1997)	Chest	346	0.04 (1.14 %)	0.11 (2.9 %)	0.11 (2.9 %)	0.03 (0.9 %)	1.70 %
Damascelli et al. (1997)	Chest	134	0.325 (6.0 %)	0.08 (1.5 %)	NA	NA	NA
Funaki et al. (1997)	Chest	80	0.57 (9.0 %)	0.08 (1.3 %)	0 (0 %)	NA	5.00 %
Koch et al. (1998)	Chest	1500	0.17 (4.8 %)	0.11 (3.2 %)	0.08 (2.4 %)	0.02 (0.67 %)	11.90 %
Biffi et al. (1999)	Chest	68	0.10 (2.8 %)	0 (0 %)	0 (0 %)	NA	2.80 %
Lorch et al. (2001)	Chest	125	0.2 (2.4 %)	0 (0 %)	0.09 (0.8 %)	0.18 (2.0 %)	4.80 %
Deppe et al. (1996)	Upper arm	154	NA (7.1 %)	NA (3.2 %)	NA	NA	3.89 %
Hata et al. (1998)	Forearm	104	0.657 (5.8 %)	0 (0 %)	0.22 (1.9 %)	0.22 (1.9 %)	5.80 %
Bodner et al. (2000)	Upper arm	109	0.38 (9.9 %)	0.17 (4.5 %)	0 (0 %)	0.45 (11.7 %)	18.00 %
Burbridge et al. (2000)	Upper arm	125	0.12 (3.2 %)	0.15 (4.0 %)	NA	0.09 (2.4 %)	NA
Tsuboi et al. (2003)	Forearm	1350	0.26 (2.9 %)	0.08 (0.9 %)	0.02 (0.2 %)	0.15 (1.6 %)	5.90 %
Mori et al. (present study)	Upper arm	433	0.17 (9.9 %)	0.04 (2.3 %)	0.04 (2.3 %)	0.04 (2.3 %)	16.63 %

*NA* not available

The incidence of CVP-related infection (catheter infection and port pocket infection) was low at 0.17/1000 catheter days (10.0 %), although the routine administration of prophylactic antibiotics was not standard in this study. Since the proper usage of antibiotics in the management of CVPs has not been proposed yet, further prospective randomized studies are warranted to determine whether prophylactic antibiotics can effectively reduce the incidence of CVP-related infection. Regarding the management of CVP-related infections, the port system should be removed in cases of suspected bacteremia or confirmed bacteremia/fungemia, along with the prompt commencement of proper antibacterial and/or antifungal treatment [28]. In cases of port pocket infection without any signs of bacteremia, port removal is not necessary for improvement of the infection, which can be treated by antibiotic therapy as in 2 out of 12 cases of port pocket infection in this study.

With regard to the incidence of venous thrombosis, it was speculated that the longer intravascular catheter of an arm CVP system could increase the risk of venous thrombosis [29]. However, Marcy et al. argued that catheter-related venous thrombosis was not associated with catheter length [30, 31]. In accordance with their argument, an extensive analysis of forearm CVPs in a large cohort of patient’s [27] as well as in our study actually demonstrated a lower incidence of venous thrombosis (Table 3). Regardless of the indwelling site of the CVPs, the prophylactic administration of anticoagulants to prevent venous thrombosis is not recommended according to the Standards, Options and Recommendations (SOR) [32] and other guidelines [33]. However, it is well known that cancer patients are predisposed to thromboembolic diseases, and that chemotherapy can also raise the risk of thrombosis [34, 35]. In this study, asymptomatic venous thrombosis was detected in 7 out of the 10 patients, thereby preventing future life-threatening thrombosis. Therefore, one should pay attention to the development of asymptomatic thrombosis on regular computed tomography examinations in patients with UACVPs, regardless of the indwelling length (Fig. 3). In addition, although Doppler US is superior to computed tomography in detecting venous thrombosis [36], we did not employ Doppler US in routine practice for the detection of venous thrombosis during the study period. Therefore, there was a possibility of underestimating the incidences of catheter-related thrombosis.

We examined whether the Bmab-containing CTx group had a tendency to develop venous thrombosis or not. According to a large pooled analysis of 6,055 cancer patients, the addition of Bmab to chemotherapy did not statistically significantly increase the risk of venous thromboembolisms compared to chemotherapy alone [37]. In our study, no significant difference was observed in the incidences of venous thrombosis between the Bmab-containing CTx group and the Bmab-free group (*p* = 0.4119). However the frequency of CVP–related complications tended to be higher in the Bmab-containing CTx group compared to the Bmab-free group (*p* = 0.022). Since the usage of CVPs was not evaluated in the pooled analysis, additional investigation is required to evaluate the effects of Bmab on the incidence of venous thrombosis and CVP-related complications.

The incidence of catheter dislocation in our study (2.1 %, 0.036/1000 catheter days) was compatible to that of previous reports [23–27]. Although we could not clarify the mechanisms responsible for the dislocation, extreme motion of the upper extremities might induce a migration of the catheter tip into the innominate or subclavian veins. Since a catheter dislocating into narrow veins can cause venous thrombosis, the catheter should be removed even when patients remain asymptomatic. Furthermore, if the patient complains of chest or back pain at the initiation of systemic administration or if catheter dysfunction occurs, the tip position of the catheter should be immediately evaluated by chest radiography or fluoroscopy with contrast medium. These precautions can prevent serious complications due to catheter dislocation. In ours study, no serious complications associated with catheter dislocation have occurred so far.

In our study, there were four cases of skin dehiscence and two cases of port chamber twist. In order to minimize skin dehiscence, it seemed important to implant the UACVPs under the subcutaneous fat tissue, not just under the dermis. In one case of port chamber twist, the port was not fixed with any sutures. In the other case, the reason why the port was inverted in the small pocket remained unresolved. It would be desirable to fix the port with a few sutures in a pocket which just fits the size of that port.

UACVPs are well accepted, especially by female patients, in terms of more convenient CTx since they do not have to get undressed for the insertion or removal of a needle, thereby relieving the embarrassment of undressing. In addition, UACVPs can be easily covered with a short-sleeved shirt. Since UACVPs are generally smaller than chest CVPs, one can experience some difficulty in inserting the needle into a smaller puncture area of the UACVPs, especially in obese patients. The self-insertion of a needle is also sometimes difficult, since patients with UACVPs cannot hold the CVPs by themselves when they insert the needle. Therefore, UACVPs seem unsuitable for long-term total parenteral nutrition in patients with short-bowel syndrome and other gastrointestinal disorders, although the short-term usage of UACVPs can be useful for fluid supplementation in advanced cancer patients as in the non-CTx group.

In conclusion, UACVPs seem to be feasible especially for systemic CTx in terms of port availability, and could be one of the options for CVP implantation in patients with some difficulties in accessing their subclavian or internal jugular veins. However, since this is a retrospective study with some limitations, a randomized clinical trial comparing the safety and utility between UACVPs and chest CVPs (including internal jugular vein access) is warranted.
